# Revising nursing home design standards and regulations in the context of an aging population: insights from smart care buildings

**DOI:** 10.3389/fpubh.2025.1540058

**Published:** 2025-10-22

**Authors:** Xiao Teng, Zhenjiang Shen, Yuntian Zhang, Arisa Ueno, Akira Murata

**Affiliations:** ^1^Department of Geosocial Fundamental Sciences, Faculty of Science and Engineering, Kanazawa University, Kanazawa, Japan; ^2^Faculty of Transdisciplinary Sciences, Institute of Philosophy in Interdisciplinary Sciences, Kanazawa University, Kanazawa, Japan; ^3^International Joint Laboratory of Smart Habitat for Humanity, Fuzhou University, Fuzhou, Japan

**Keywords:** smart device, older population care facilities, smart care, regulatory, demand analysis

## Abstract

In this study, based on the use of smart devices in nursing home, recommendations are made for optimizing existing regulations and standards in Japan. Japan’s housing standards and legal regulations for older population in the context of aging are reviewed firstly, existing regulations and standards are summarized to meet the demands of older population’s housing; the selection of smart medical devices that will effectively be introduced will be based on the demands of older population’s housing. The smart medical devices proposed in this study can be integrated with Japan’s older population’s housing model to enhance the content of existing regulations. Ultimately, a method and a list of improvements to current regulations based on the use of smart devices is proposed. With improved of the regulations, it is possible to eliminate the problem of shortage of personnel in nursing homes, improve work efficiency, and promote cooperation between facilities and care staffs.

## 1 Introduction

Smart care is taking on a revolutionary role in the field of care for the older population by integrating advanced technologies and smart systems to provide more personalized, comprehensive and efficient care for the older population (1). In the field of nursing home for the older population, there is also a next generation housing model called the “smart care model” that uses smart devices from the Internet of Things (IoT) and information and communication technology (ICT) in smart buildings to address the problems of a super-aging society (2, 3).

**Figure 1:**
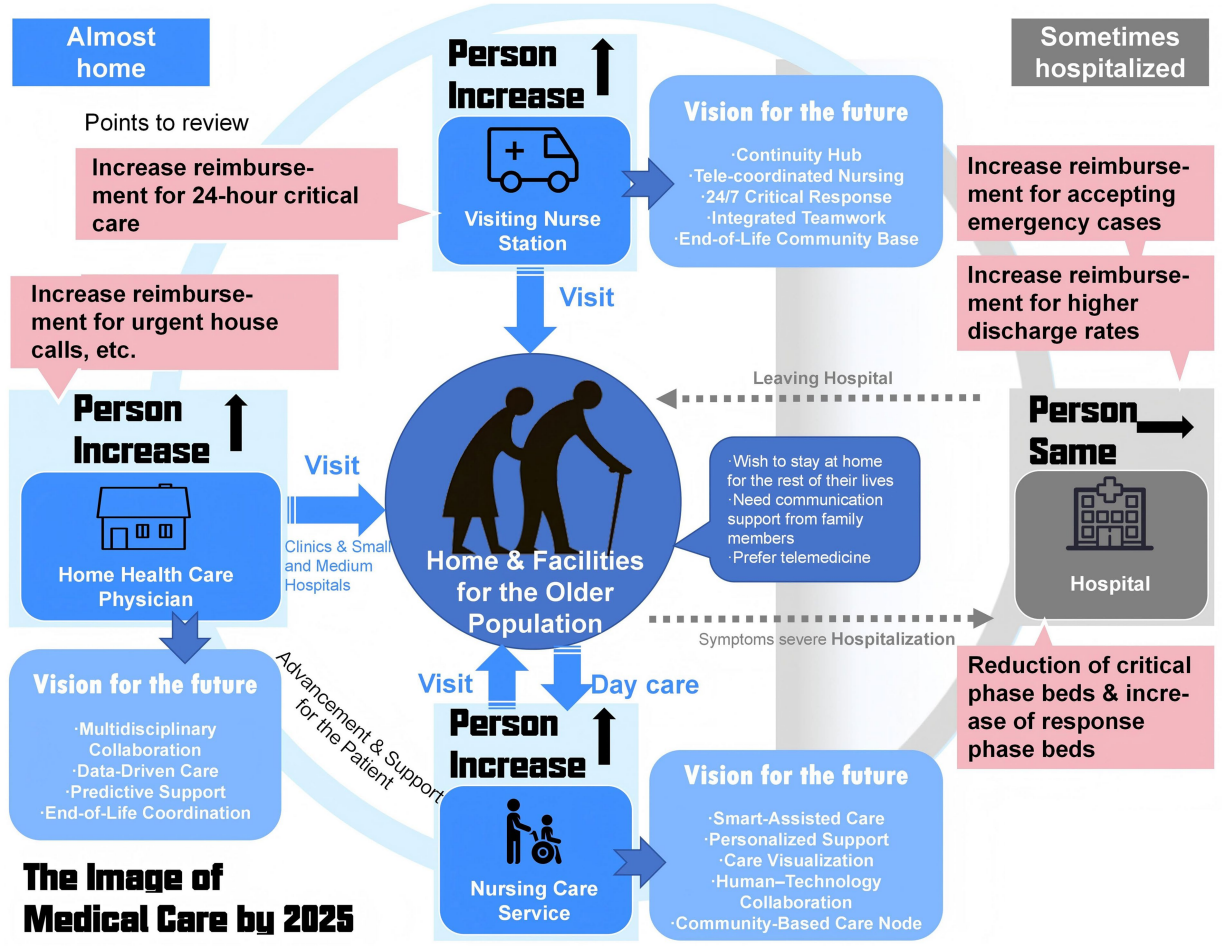
Near-future trends in Japan’s older population facilities.

The “Japan 2025 Problem (Hyper-Aging Society)” mentions that as the number of older population increases, the number of people in demand for nursing and medical care will also increase (4), resulting in two types of problems: “the burden of social security costs will become heavier” and “it will become difficult to maintain the medical and nursing care system” (Figure 1). The use of smart devices in the “smart care model” can promote preventive medicine and self-care, as well as information sharing between users and health and welfare specialists by providing feedback to users through biometric data accumulated through the use of smart devices (5–7). This not only improves convenience but also improves the health of the population, which is considered one of the issues in “Japan’s 2025 Problems,” and the use of smart devices is intended to reduce the pressure on medical care and nursing care that will arise in the future (8).

Existing research on “smart care” discusses the effectiveness of home health care and smart devices in an aging society, including the use of technologies such as smart wearable devices, telemedicine services, smart pill boxes, home environment monitoring and mattress and bedside monitoring (9–12). However, there is a lack of research in the field of preventive medicine for early detection before the demand for care arises, and there has been no significant progress in building systems that are effective for primary and secondary prevention in particular, where primary prevention focuses on preventing disease in healthy individuals through lifestyle changes, health education, and vaccination, and secondary prevention involves early detection and timely treatment to avoid disease progression, typically targeting those who may already be affected but show no symptoms (13, 14). In order to provide personalized services in the future, systems that incorporate preventive medicine, such as disease prevention and early detection, will be required (15). Although the “smart care model” currently promotes the use of information and communication technologies, it is limited to the use of medical devices, and aims to use information and communication technologies to transmit information obtained from medical devices, and the specific design of smart housing with integrated smart devices is still emerging, with limited reference cases or established regulations available (16, 17).

At present, most available reference cases are found in independent housing, such as those discussed in the shift from smart homes to smart-ready homes and communities. For example, the TigerPlace project in the U. S. realizes home health monitoring and remote care through a sensor network (18); MaaR Housing in the Netherlands provides modular senior housing and links with community medical facilities (19); the Kashiwanoha Smart City in Chiba Prefecture, Japan, combines IoT and AI technologies to provide smart health management and housing support for the older population (20); and the CARE-WISE project in the U. K. focuses on the use of smart devices for the older population living alone (21). However, these examples tend to focus on general residential contexts and offer limited guidance for older population-oriented smart housing design.

Globally, countries have established older population’s care design standards tailored to their demographic aging challenges. In the United States, the ADA Accessibility Guidelines ensure barrier-free environments through clear regulations on spatial accessibility and assistive device placement, widely used in senior housing and medical settings (22). Canada’s long-term care system combines federal and provincial standards, with CSA Z8004 and Z2004 addressing safety and mental health (23, 24), while laws like the Long-Term Care Homes Act regulate operations, service quality, and older population’s rights (25). Europe promotes the “universal design” concept via ISO 21542:2021, focusing on safety, autonomy, and social interaction within both residential and community spaces (26).

Singapore’s eldercare system is family-based with community and institutional support, regulated by the Healthcare Services Act and Nursing Home Service Regulations, which set licensing and care standards (27, 28). South Korea’s system centers on LTCI, with the 2024 Community Care Assistance Act enhancing integrated community care and staffing guided by the Welfare of the Aged Act (29, 30). China’s GB50340-2016 standard details requirements for living, public, and medical spaces in older population’s care facilities, emphasizing functional age-friendliness and integrated caregiving (31). Despite these efforts, a common limitation across countries lies in the predominant focus on physical accessibility and safety, while regulatory frameworks remain underdeveloped in addressing cognitive impairment, digital assistive technologies, and personalized care needs - failing to fully adapt to the increasingly diverse health and lifestyle requirements of aging populations.

As for Japan, current Japanese regulations and standards related to the living environment and nursing home for the older population are mainly based on accessibility and barrier-free access, safety standards, health-care facilities, social and activity spaces, energy-saving and environmental protection requirements, and the demand for personalized care, as well as staffing requirements, registration and specific design requirements for nursing home for the older population. However, with the advancement of technology, the current regulations may not adequately cover the use of emerging technologies; at the same time, due to the rapid changes in technologies, the design of the regulations should have a certain degree of flexibility and be able to adapt to new technologies and situations that may arise in the future (32, 33). It is thus necessary to review and adjust the relevant legal regulation to ensure that the regulations keep pace with technological development (34).

Therefore, in this study, the existing regulations and standards in Japan were revised based on the selection of smart devices in accordance with the demands of the older population in the next generation. Firstly reviewed the documents, summarized the requirements for older population housing in regulations and standards in Japan. Based on the evaluation of smart devices, the demands of the next generation of housing for the older population were presented and the selection of smart medical devices that will actually be introduced was presented. The impact of the use of smart devices on the relevant regulations and standards and the improvement of the content of the regulations due to their impact are finally summarized, and optimization proposals are made. The optimized regulations and standards can be used as a basis for the preparation of documents related to smart care buildings in the absence of examples of existing regulations and standards that can be used as a reference for the construction of smart care buildings for the time being.

## 2 Methodology

**Figure 2:**
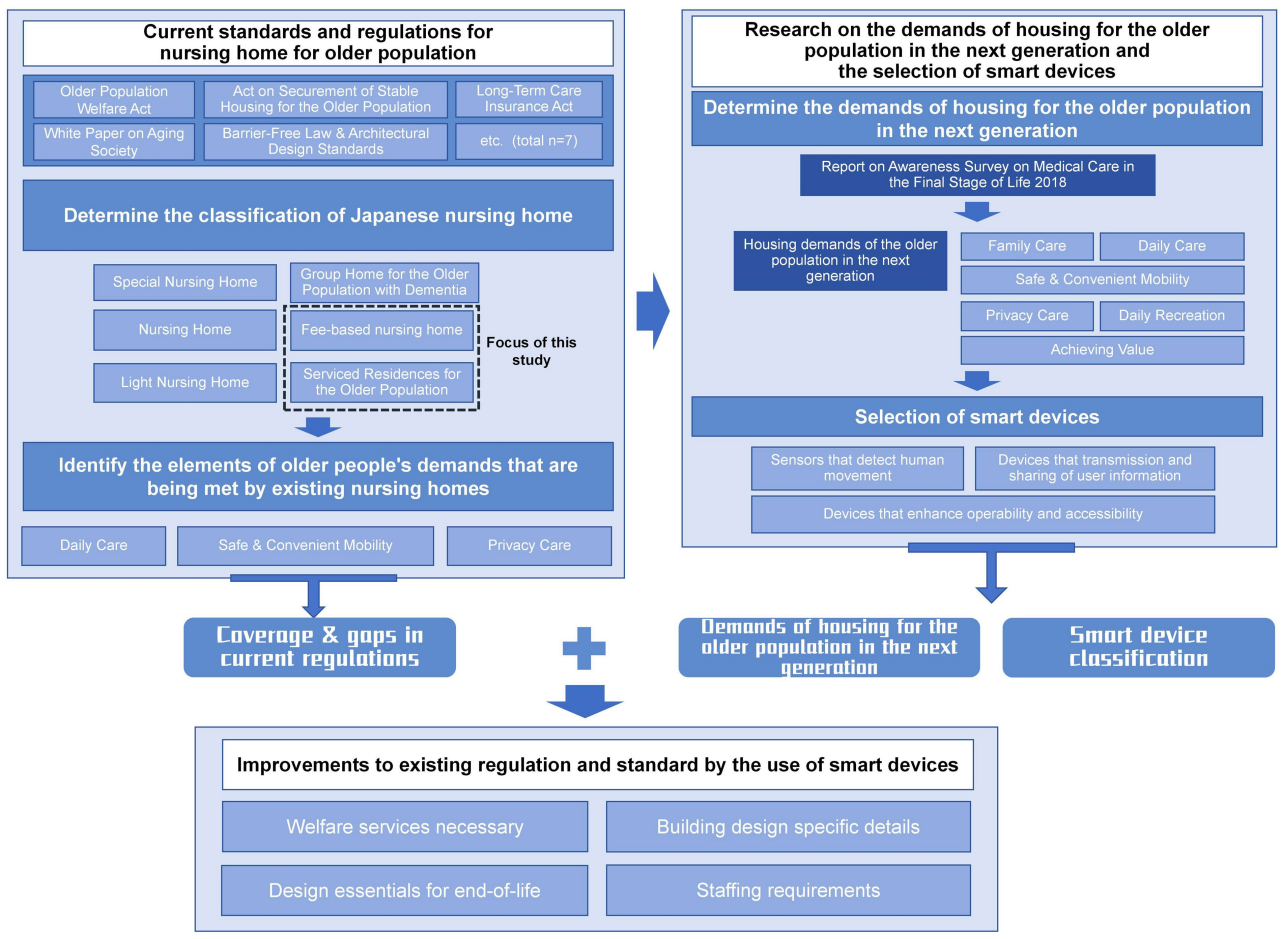
Research processes.

In order to satisfy the demands of the older population in the context of aging, this study argues that the documents related to the design of smart care buildings need to clarify the current living environment of the older population and the older population’s housing-related technology, analyze the existing regulations and summarize the standards of older population’s housing, and, based on the choice of smart devices and the characteristics of the smart devices, to put forward improvement suggestions to the current standards of design of nursing home and the law (Figure 2).

I: Review existing regulations for older population’s housing in Japan, summarize design and configuration requirements, and analyze how current regulations meet older population’s housing needs to inform smart device selection and regulatory improvements.

Phase I focuses on clarifying the framework and limitations of existing standards, answering the question of “where the current system stands.” This phase reviews key Japanese laws related to older population’s housing, including the Older Population Welfare Act, the Act on Securement of Stable Housing for the Older Population, and the Long-Term Care Insurance Act, as well as relevant design standards such as the Barrier-Free Law and Architectural Design Standards, and policy documents like the White Paper on Aging Society. This phase summarizes the main concerns of current regulations, design guidelines and policy documents related to senior housing (Table 1), focusing on functionality, safety and care efficiency, as well as privacy protection and humanistic care, respectively, to provide a basis for the subsequent selection of smart devices and system optimization. This phase highlights both the scope and the limitations of the current framework, laying the groundwork for subsequent smart device selection and regulatory improvement.

**Table 1:** Overview of different aspects of analysis.

Element category	Key analytical focus
Existing older population’s housing regulations	Types and functional positioning of older population’s housing
	Legal basis and applicable populations
	Care efficiency–related requirements
	Mobility safety–related requirements
	Infrastructure and utility safety
Existing design guidelines for older population’s housing	Care efficiency–related requirements
	Mobility safety–related requirements
	Infrastructure and utility safety
Existing aging-friendly construction policy documents	Privacy and humanized care
	Basic living support spaces
	medical support and safety
	Emotional care and family interaction

The study first clarifies the applicable contexts and content types covered by each regulation and standard, including aspects related to spatial layout, safety measures, service processes, and equipment or facility design and operation. Second, it identifies mandatory indicators specified in current regulations and standards—such as corridor widths, door clearances, handrail installations, accessible restroom configurations, and staff-to-resident ratios. Finally, the study summarizes the older population’s housing needs currently addressed by regulations and standards into three categories: daily care support, safe and convenient mobility, and privacy protection (Figure 2). This phase identifies both the scope and limitations of existing regulations, laying the groundwork for the subsequent selection of smart devices and regulatory improvement.

II: Propose next-generation older population’s housing requirements based on I, clarifying current regulations, identifying demand similarities and differences, and evaluating smart devices for a demand-driven approach.

Phase II focuses on the coupled needs of a super-aged society and smart care technologies, answering the question of “what future developments require.” Based on the coverage and limitations of current Japanese regulations, this phase incorporates data from the 2018 nationwide public awareness survey conducted by the Ministry of Land, Infrastructure, Transport and Tourism to identify the core housing needs of future older population. These needs are categorized into six domains: Family Care, Daily Care, Safe and Convenient Mobility, Privacy Care, Daily Recreation, and Achieving Value. The study selects individuals aged 20–39 as representatives of the older population in 2050, and constructs a framework for “next-generation older population’s housing needs” based on their behavioral characteristics and demand preferences.

Subsequently, the study proposes an “initial decision pathway” approach, integrating current regulatory provisions with practical caregiving practices and employing a dual intervention strategy of spatial layout optimization and smart device integration to respond to each need category. On this basis, smart devices are classified into three functional types—sensors that detect human movement, devices that transmission and sharing of user information, and devices that enhance operability and accessibility. Their spatial deployment is analyzed. These findings offer technical support and policy recommendations for future regulatory revisions and smart device selection. This phase provides technical evidence and policy recommendations for demand-driven improvements to current standards.

III: Improve current older population’s nursing home regulations based on smart device types and characteristics from II, summarizing their impact on regulations and proposing optimizations for smart care building-related documents.

Phase III builds upon the findings of Phases I and II to propose targeted pathways for revising current standards, answering the question of “how institutional improvements can be realized.” This phase builds upon the Japanese government’s classification of six core older population’s welfare services, identifying four of them as services that can be realized through smart devices, and reorganizes them under a new category of “smart services.” The study further maps these smart services to the 6 key housing needs identified in Phase II, proposing strategies for optimizing architectural standards based on device functionalities. Finally, the study evaluates the impact of smart devices on staffing requirements in fee-based older population, concluding that remote monitoring and behavioral data collection can reduce the burden of nighttime patrols and manual recordkeeping. This approach demonstrates how smart technologies can enhance service efficiency, alleviate staffing shortages, and provide concrete technical support for institutional reform and smart care policy development.

This study follows a three-phase framework: Phase I clarifies the framework and limitations of current older population’s housing regulations, answering “where the system stands”; Phase II identifies future needs by coupling aging society demands with smart care technologies, answering “what future development requires”; Phase III builds on these findings to propose targeted revisions, answering “how improvements can be realized.” Together, the three phases form a coherent progression from diagnosis of the current system to projection of future demands and finally to actionable pathways for regulatory reform.

## 3 Results

### 3.1 Organization of current standards and regulations for nursing home for older population

Based on the study of Japan’s Older Population Welfare Act, Act on Securement of Stable Housing for the Older Population, and Long-Term Care Insurance Act, it is clear that the existing regulations and policies classify nursing homes into Special Nursing Home, Nursing Home, Light Nursing Home, Group Home for the Older Population with Dementia, Fee-based nursing home and Serviced Residences for the Older Population. For the subject of this study, which is mainly a home or service housing for the older population with no special demands and accessible to all types of older population, this study will mainly analyze the design standards and the requirements of older population in the two major types of older population’s homes: Fee-based nursing home and Serviced Residences for the Older Population. In particular, Fee-based nursing home can be further subdivided into three subcategories (Table 2), according to which the staffing standards of Fee-based nursing home are different (35–37).

**Table 2:** Overview of regulation-based homes for the older population without special demands.

Type of nursing home (major category)	Type of nursing home (minor category)	Definition by regulations	Suitable types of older population
Fee-based nursing home	Nursing Care fee-based nursing home	Residential facilities for the older population with nursing care and other services	Residences that provide nursing care and other services for the older population and are open to all types of older population.
	Residential fee-based nursing home	Residential facilities for the older population with services such as daily living assistance	All types of older population can be admitted, and facilities with services such as support for daily living can provide nursing care and other services (nursing care can be withheld if not necessary).
	Health-type fee-based nursing home	Residential facilities for the older population with meals and other services	Residences for all types of seniors, mainly for seniors who do not need nursing care
Serviced residences for the older population	-	Section 5 of the Act on Securement of Stable Housing for the Older Population is defined as housing that provides welfare services such as condition monitoring services and lifestyle counseling services.	Over 60 years old or single/couple families under 60 years of age who need care and nursing care

Based on the review of regulations, the study summarizes the housing demands of the older population that are satisfied by the existing regulations related to housing for the older population (Table 3). The current demands of the older population that are based on daily care, safe and convenient mobility, privacy care. The concept of the next-generation older population was proposed by the Ministry of Health, Labor and Welfare of Japan in its “Vision 2035.” According to Japan’s White Paper on the Aging Society and the Council for Designing the 100-Year Life Society (38, 39), the demands of the next-generation older population should at minimum include end-of-life care, independent living, emotional connection and social interaction, diversity and personalization, and preventive, health-oriented management. The three categories of current demands are not able to cover all the demands of the next generation older population. Therefore, there is a clear understanding of the housing demands of the next generation of older population that cannot be satisfied by traditional design guidance and regulation requirements.

**Table 3:** Housing demands of older population that are satisfied by the existing regulations.

Type of demand	Content of the demand	Methods of satisfying the demand
Daily care	Efficient and reasonable care	1. The older population are categorized into levels of care and are cared for according to the level of care; 2.different levels and types of nursing homes are equipped with different numbers and types of caretakers
Safe and convenient mobility	Reasonable scale	1. Home facilities such as steps, hand-washing stations, bathtubs, electric switches, etc. are set at a reasonable scale according to the mobility range of the older population; 2. Passageways and entrances and exits in rooms are set at a reasonable scale according to the mobility range of the older population
	Safe use of water and electricity	Water supply and hot water devices, electrical devices and gas devices are equipped with safety devices
	Ensuring indoor light	The light intensity of lighting devices is limited
Privacy care	Privacy care in daily life	Installation of undressing facilities and specialized shoe removal areas at entrance halls

### 3.2 Research on the demands of housing for the older population in the next generation and the selection of smart devices

To address the next-generation older population needs explicitly outlined in Japan—such as end-of-life care, independent living, emotional connection and social interaction, diversity and personalization, and preventive, health-oriented management—this chapter identifies specific housing requirements for the future older population based on a national public awareness survey. The survey, titled “Report on Awareness Survey on Medical Care in the Final Stage of Life,” was published by the Ministry of Land, Infrastructure, Transport and Tourism of Japan in 2018 (40).

The survey was conducted nationwide by the Japanese government, targeting general residents aged 20 and above, and yielded 3,000 valid responses. A stratified sampling method was used based on gender, age, and region, covering all prefectures to ensure national representativeness. The sample design aimed to capture public perceptions and attitudes toward older population’s medical care, end-of-life support, and residential choices, serving as a foundation for future policymaking. Given the projected needs of the next-generation older population, this study focuses on a specific age group of 20–39 years old, representing those who will be aged 60 and over by 2050. A total of 879 valid samples from this group were analyzed.

**Figure 3:**
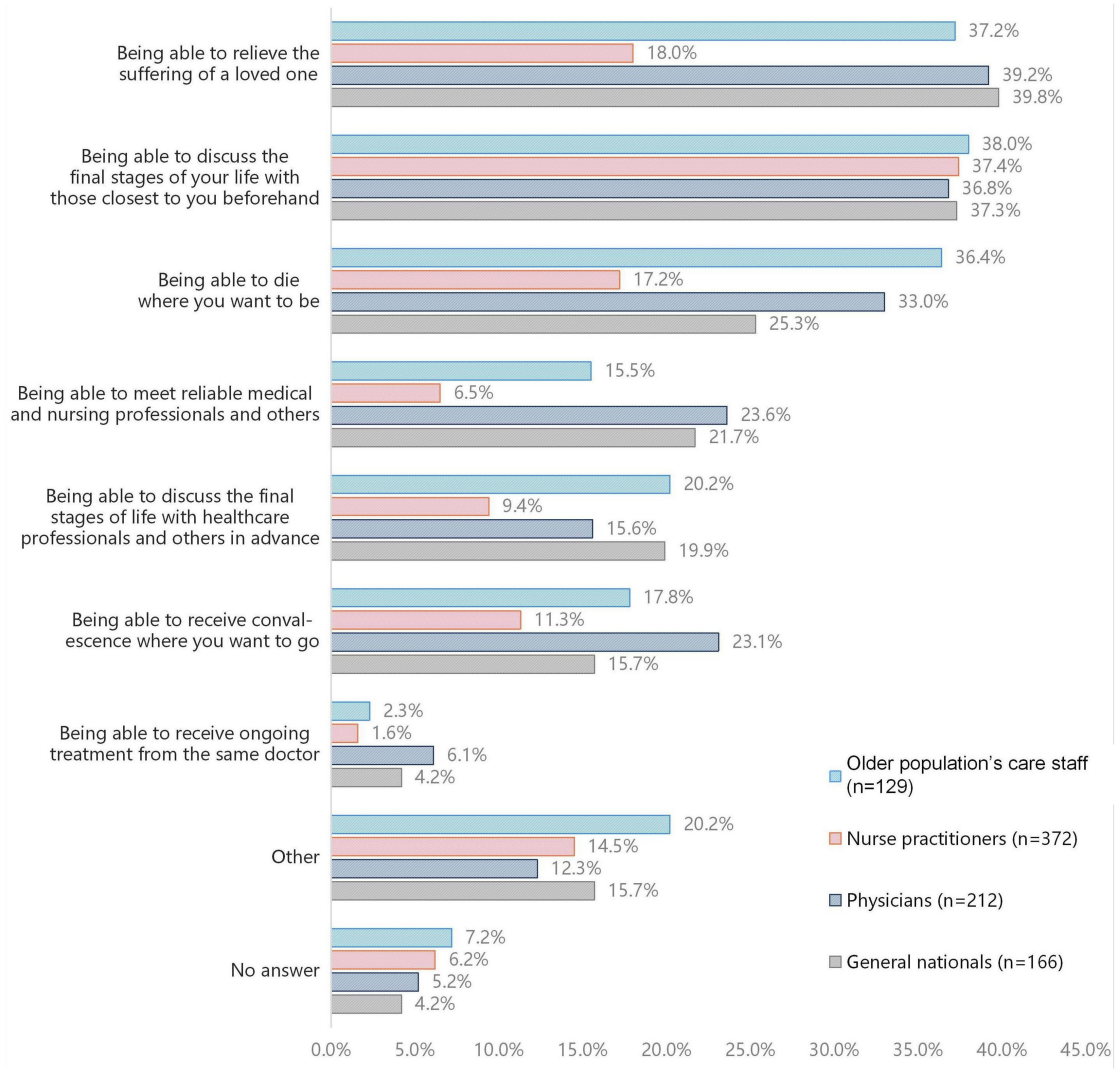
Survey data on practices that would be taken to avoid end-of-life regrets (Compiled from the Report on Awareness Survey on Medical Care in the Final Stage of Life published by the Ministry of Land, Infrastructure, Transport and Tourism of Japan).

According to the results, the most suitable place for hospice care is a place where adequate medical and nursing services are available and where people have family members with them (Figure 3).

Therefore, when designing housing for the older population, the first step is also to identify the items necessary for the design of housing and end-of-life home care for the older population based on the survey data (Table 4).

**Table 4:** Requirements for designing housing for the older population and end-of-life home health care (Compiled from the Report on Awareness Survey on Medical Care in the Final Stage of Life published by the Ministry of Land, Infrastructure, Transport and Tourism of Japan).

No.	Purpose	Content in detail
1	Ease of monitoring	Easy for family members to monitor the patient’s condition
2	Ease of care	Efficient care is possible
3	Ability to spend time with family	The patient can spend more time with the family
4	Compact recuperation space	Activities of daily living can be completed within a small area
5	Maintaining living functions	The function of the living room can be maintained as a common space for serving guests.
6	Continuity of living arrangements	The original lifestyle can be maintained.
7	Securing privacy	Privacy can be maintained for visitors.
8	Access to restrooms	Easy access to the toilet from the treatment space
9	Access to the outside	Easy access to the outside from the medical care space
10	Barrier-free	Safety of movement is ensured, with no steps or obstacles

Based on the collation of the content of the regulation and hospice care, the study summarizes the housing demands of the older population in the next generation based on the behavioral characteristics and living requirements of the older population (Table 5). “Initial decision pathway” is based primarily on the design techniques mentioned in the existing design standards (41, 42), and secondly on the design techniques used in actual cases in Japan (43–45). These decisions reflect realistic and applicable solutions, including spatial planning strategies and the incorporation of smart technologies, to address the behavioral characteristics and care-related needs of next-generation older population. Each “Initial decision” not only indicates the use of smart devices but also specifies the category of device, aiming to guide the subsequent selection of specific equipment. At the same time, it incorporates “rational spatial layout” (41), emphasizing a strategy of combined physical and technological intervention to avoid isolated reliance on technology. The analysis of these demands highlights the pivotal role of smart devices in meeting the increasingly complex and diverse requirements of older population. Integrating such technologies into housing environments is fundamental to expanding the capacity of current care systems and providing targeted, efficient solutions to age-related living issues.

**Table 5:** Housing demands of the older population in the next generation with initial decision pathway.

Types of demand	Content of demand	Initial decision pathway
Family care	Easy for family members to monitor the patient’s condition	Using smart devices (monitoring sensor devices, data transmission devices)
	The older population can spend more time with family members	Use of smart devices (communication devices, data transmission devices)
Daily care	Efficient care	Rational floor plan, efficient care flow, use of smart devices
	Rapid access to physical data of the older population	Use of smart devices (monitoring sensor devices)
	Care data recording and statistics	Use of smart devices (data analysis devices, data transmission devices)
Privacy and security	Lifestyle can be maintained	Rational floor plan layout (Barrier-free/Short circulation paths/Zoned layout/Adaptable/Smart-device ready), layout of daily equipment
	Privacy is maintained when there are visitors	Rational floor plan layout (Barrier-free/Short circulation paths/Zoned layout/Adaptable/Smart-device ready), use of smart devices (smart curtains)
Safe and easy mobility	Easy access from the treatment area to the restroom	Rational floor plan layout (Barrier-free/Short circulation paths/Zoned layout/Adaptable/Smart-device ready)
	Easy access from the treatment area to the outside	Rational floor plan layout (Barrier-free/Short circulation paths/Zoned layout/Adaptable/Smart-device ready)
	Mobility safety is assured without steps or obstacles	Rational floor plan layout (Barrier-free/Short circulation paths/Zoned layout/Adaptable/Smart-device ready), use of smart devices (smart curtains)
	Activities of daily living can be accomplished in a small area	Rational floor plan layout (Barrier-free/Short circulation paths/Zoned layout/Adaptable/Smart-device ready), layout of daily devices, use of smart devices
	The function of the living room is maintained as a communal and reception space	Rational floor plan layout (Barrier-free/Short circulation paths/Zoned layout/Adaptable/Smart-device ready), use of smart devices
	Bathroom and toilet facilities are easily accessible	Rational floor plan layout (Barrier-free/Short circulation paths/Zoned layout/Adaptable/Smart-device ready), efficient life flow, use of smart devices
Daily recreation	Daily recreation with other seniors, family members, etc.	Rational floor plan layout (Barrier-free/Short circulation paths/Zoned layout/Adaptable/Smart-device ready), use of smart devices (remote interactive devices, AR, VR, etc.), rational community planning
Achieving value	Participation in creative activities and realization of the social value of the older population	Use of smart devices (smartphones, AR, VR, etc.), rational community planning

Currently available smart medical and assistive devices on the market can be broadly categorized into three functional types based on ISO 9999:2022 and ISO/IEC 30141:2018: (1) Sensors that detect human movement (Human monitoring), (2) Devices that transmission and sharing of user information (Transmission and sharing of information), and (3) Devices that enhance operability and accessibility (Accessibility and operability) (46, 47). The first category is primarily used to monitor vital signs, sleep quality, and activity levels of older population, including wearable devices, user-operated communication tools, and environment-embedded sensors (48). The second category leverages IoT technology and smartphone platforms to enable real-time data sharing and remote collaboration between the older population and their caregivers or family members (49). The third category improves user experience through voice control, app-based interfaces, and other features that support independent living for seniors with visual or physical limitations (50, 51).

Table 6 illustrates how these three types of smart devices work together as an integrated and efficient system within residential environments. Sensors are responsible for data acquisition, continuously capturing physical condition indicators of older population through wearable devices, user-operated tools, or embedded environmental sensors. The collected data is then transmitted via connectivity devices that link to IoT platforms, enabling real-time cloud-based data sharing and access across multiple terminals. This seamless data transmission supports continuous monitoring and timely response. Operability and accessibility devices, strategically installed in areas such as bedrooms, bathrooms, kitchens, and living rooms, optimize user interaction and assistive functionality. Together, these components—data acquisition, transmission, and efficiency-enhancing technologies—form a cohesive system that enhances the quality, responsiveness, and continuity of older population’s care.

**Table 6:** A combination of smart devices and services based on the housing demands of the older population in the next generation.

Location	Type of smart device/service	Elements that could be included	Satisfied demands
Bedroom	Air conditioner	Accessibility and operability	Maintaining the lifestyle originally intended
	Light bulb	Accessibility and operability	Maintaining the lifestyle originally intended
	Ceiling lights	Accessibility and operability	Maintaining the lifestyle originally intended
	Electric reclining beds	Accessibility and operability	Maintaining the lifestyle originally intended
	Mattresses	–	Maintaining the lifestyle originally intended
	Sleep quality analysis sensor	Human monitoring, transmission and sharing of information	Easy monitoring of the patient’s condition by family members, quick access to physical data of older population
Living room	Monitoring service	Human monitoring, transmission and sharing of information	Efficient nursing care, quick access to physical data of the older population, and recording and counting of nursing care data
	Smart speakers	Accessibility and operability	Daily recreation with other older population, family members, etc.
	Streaming services	Accessibility and operability	Daily recreation with other older population, family members, etc.
	Televisions	Accessibility and operability	Daily recreation with other older population, family members, etc.
	Robot vacuum cleaners	Accessibility and operability	Efficient care
	Smart windows	Accessibility and operability	Privacy is maintained when there are visitors, maintaining the lifestyle originally intended
	Air conditioners	Accessibility and operability	Maintaining the lifestyle originally intended
Bathroom and dressing room	Washers and dryers	Accessibility and operability	Easy to use bathroom and toilet facilities
	Water Heaters	Transmission and Sharing of Information, Accessibility and operability	Easy to use bathroom and toilet facilities
	Remote controls for water heaters and heat source units for hot water heating	Transmission and sharing of information, accessibility and operability	Easy to use bathroom and toilet facilities
	Electric bath ventilation dryers	Accessibility and operability	Easy to use bathroom and toilet facilities
	Weight scales	Transmission and sharing of information, accessibility and operability	Easy for family members to monitor the patient’s condition and quickly obtain data about the older adult’s health.
Kitchen	Microwave ovens	Accessibility and operability	Maintaining the lifestyle originally intended
	Refrigerators	Accessibility and operability	Maintaining the lifestyle originally intended
Entrance	Door camera kit with monitor	Transmission and sharing of information, Accessibility and operability	Privacy is maintained when there are visitors
	Smart locks	Accessibility and operability	Privacy is maintained when there are visitors

By optimizing human-device interaction, these tools promote independence and ease of use in daily life, particularly for those with mobility limitations or sensory impairments. The combined application of these three device categories across living spaces reflects a systematic integration of smart technologies in older population’s housing. This approach not only improves functionality but also addresses the increasingly diverse and complex living and care needs of the next-generation older population.

Through the use of the summarized smart devices, it is possible to realize some of the demands of the older population that cannot be realized by traditional devices, and to provide more of the services that are required to be provided in a nursing home. As a result, some of the provisions in regulations and standards will be adjusted due to the addition of smart devices. Based on the summarized smart devices, the study will propose regulations and standards that can be improved and the specific provisions improved modalities.

### 3.3 Improvements to existing regulation and standard by the use of smart devices

According to the definitions of the Ministry of Land, Infrastructure, Transport and Tourism and the Ministry of Health, Labor and Welfare, six services are classified as welfare services necessary for the daily lives of the older population: “Condition monitoring services,” “Lifestyle counseling services,” “Nursing care-related services such as bathing,” “Meal-supply-related services,” “Household-related services, such as cooking, washing and cleaning,” and “Maintenance and promotion of physical and mental health.” Four of these services: “condition monitoring services,” “lifestyle counseling services,” “care-related services such as bathing” and “maintenance and promotion of physical and mental health” can be realized through the use of smart devices.

These six services are independent of the type of welfare facility like nursing homes, these six services can be categorized as “smart device utilization services” and “communication services” (Table 7).

**Table 7:** Revision of welfare services necessary for the older population to lead their daily lives.

Category	Service type	Purpose/function
Smart device utilization services	Condition monitoring services	Real-time tracking of physical condition, emergency alerts
	Lifestyle counseling services	Personalized health guidance, mental support
	Household-related services, such as cooking, washing and cleaning	Reduce daily physical workload, maintain autonomy
	Maintenance and promotion of physical and mental health	Prevent decline in physical/cognitive functions
Communication services	Meal-supply-related services	Ensure meal planning and nutritional support
	Nursing care-related services (e.g., bathing, toileting assistance)	Enhance safety, efficiency, and dignity in personal care (e.g., aided by adjustable bathing lifts, smart toilets, temperature-controlled bath systems)

For older population’s essential welfare services, in terms of condition monitoring services, current standards rely on in-person visits, periodic measurements, and caregiver recordkeeping, which are time-consuming and prone to delays. The proposed revision introduces smart devices to enable real-time health tracking and emergency alerts. The rationale is to improve responsiveness and reduce caregiver workload, with the ultimate effect of ensuring that emergencies are promptly detected and addressed, thereby enhancing overall safety.

In terms of lifestyle counseling services, existing practices provide only basic health advice and lack personalized support. The revision recommends the use of AI and remote platforms to deliver tailored health guidance and psychological support. The rationale is that older population increasingly present diverse needs, which cannot be met by a “one-size-fits-all” approach. The ultimate effect is to promote both physical and mental well-being, while enhancing life satisfaction and a sense of happiness.

In terms of household-related services, current practices mainly depend on caregivers or family members, which are labor-intensive and inefficient. The revision suggests adopting smart cooking, washing, and cleaning devices. The rationale is to reduce reliance on manual labor through technological means and to improve daily convenience. The ultimate effect is to ease the physical burden on the older population and help them maintain a higher degree of autonomy.

In terms of nursing care-related services, existing practices rely on manual caregiver assistance (e.g., bathing and toileting), which is both labor-intensive and potentially undermines dignity. The revision proposes the introduction of smart toilets, adjustable bathing chairs, and temperature-controlled bathing systems. The rationale is to enhance both safety and efficiency while also providing a more dignified experience. The ultimate effect is to make care services more humane and improve the overall quality of life for the older population.

In addition, the heights of sockets and switches specified in the building design standards in consideration of the smooth movement of the older population, the disabled, and others can be eliminated due to the use of smart devices (voice-operated and smart switches), such as “Intercom/monitor set indoor height: approx. 110 cm,” “Switch/outlet (bed) set indoor height: approx. 80–90 cm,” and “Switch/outlet set indoor height: approx. 40 cm,” the above standards can be eliminated by the use of smart devices (Table 8).

**Table 8:** Revision of building design standards for smooth mobility of persons with disabilities.

Design Elements	Building design standards for smooth mobility of the older population and disabled		Building design standards for smooth mobility of the older population and disabled (revised)	
	Smoothing standard	Smoothing inducement standard	Smoothing standard	Smoothing inducement standard
Concent switches	Intercom/monitor set indoor height: approx. 110 cm		Utilize smart devices	
	Switch/outlet (bed) set indoor height: approx. 80–90 cm			
	Switch/outlet set indoor height: approx. 40 cm			
Other	Counter: approx. 70–75 cm high, depth 45 cm or more		Counter: approx. 70–75 cm high, depth 45 cm or more	

Existing regulations stipulate a uniform installation height, but they overlook spatial differences and the diverse needs of the older population. The proposed revision recommends removing certain rigid height requirements and instead adopting voice control, smart switches, and similar solutions. The rationale is to avoid a “one-size-fits-all” design and enhance adaptability. The ultimate effect is to improve design flexibility and accommodate diverse spatial layouts as well as the varied usage habits of older population.

At the same time, the five requirements set forward in the existing design standards and regulations for older population’s housing and end-of-life home care (Table 4): Ease of monitoring, Ease of care, Compact recuperation space, Maintaining living functions and Securing privacy, will always apply to the use of smart devices. For example, an electric bed can be used for “Ease of care” by allowing the individual to get up on his/her own, devices related to monitoring services can be used for “Ease of monitoring,” and smart windows can be opened and closed for “Compact recuperation space,” “Maintaining living functions” and “Securing privacy.” In addition, “Ability to spend time with family” and “Continuity of living arrangements” can be realized by communicating with family members through smart monitoring services and providing data to healthcare professionals (Table 9).

**Table 9:** Investigation of items required for the designing housing for the older population and end-of-life home health care.

Purpose	Content in detail	Example to use of smart devices
Ease of monitoring	Easy for family members to monitor the patient’s condition	Use of devices related to monitoring services
Ease of care	Efficient care is possible	Use of an electric bed
Compact recuperation space	Activities of daily living can be completed within a small area	Use of smart windows and switches
Maintaining living functions	The function of the living room can be maintained as a common space for serving guests	Use of smart windows and switches
Securing privacy	Privacy can be maintained for visitors	Use of smart windows and switches
Ability to spend time with family	The patient can spend more time with the family	Use of smart monitoring services
Continuity of living arrangements	The original lifestyle can be maintained	Use of smart monitoring services

In terms of health monitoring and family companionship, the current model relies on family members’ manual observation, which is inefficient and prone to omissions, while actual communication time between the older population and their families is limited. The proposed revision introduces smart monitoring and communication devices to enable real-time sharing of health conditions, allowing family members to keep track of the older population at any time and maintain remote interaction, thereby extending companionship and strengthening emotional connections.

In terms of care and daily convenience, the traditional model depends heavily on human labor for care efficiency, and daily activities often require relatively large spaces, making it difficult for areas such as the living room to serve both reception and daily functions. The proposed revision suggests the use of electric care beds, smart windows, and smart switches as auxiliary devices to streamline care processes and, at the same time, maintain both daily living and communal functions within limited space, thus improving convenience and independence.

In terms of privacy and safety, existing approaches mainly rely on physical partitions, which lack flexibility and cannot be adjusted instantly according to visitors or situational needs. By introducing smart windows and smart switches, spatial conditions can be flexibly adapted, ensuring privacy during visits while also enhancing overall safety and comfort.

In terms of continuity of living arrangements, traditional smart renovations often disrupt the older population’s established lifestyle, leading to adaptation difficulties and psychological stress. The proposed revision emphasizes the use of smart monitoring and environmental sensing devices to provide safety and convenience while preserving original living habits as much as possible, thereby ensuring a smooth integration of care and daily life and reducing adjustment barriers.

**Figure 4:**
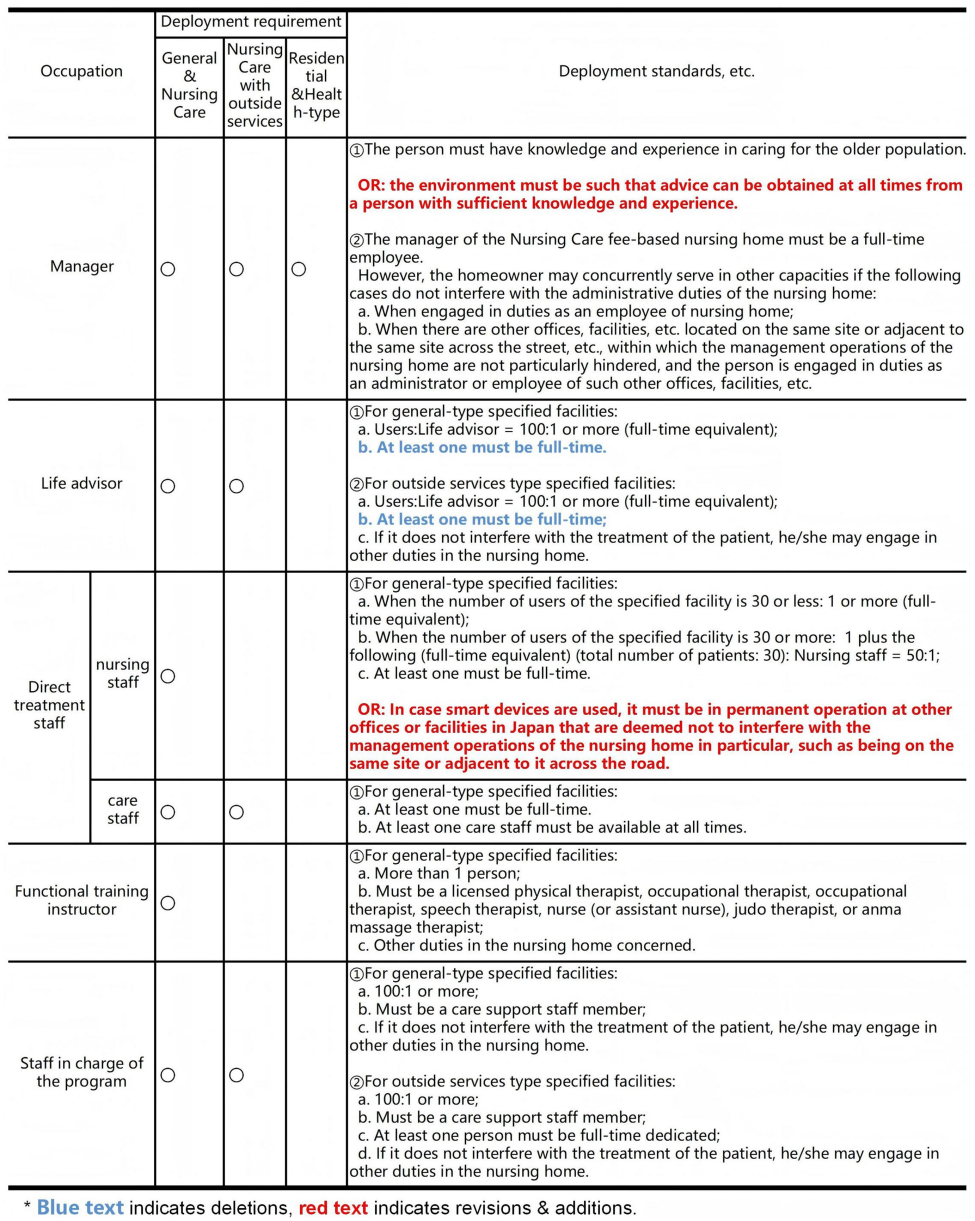
Revision of staffing standards, etc. for fee-based nursing homes.

In addition, the study concluded that the primary staffing standards for an fee-based nursing home could be modified (Figure 4). The fee-based nursing home can be either assisted or residential, and a nursing home with care normally requires a manager, a life advisor, nursing and care staff, a functional training instructor, and a staff in charge of the program. Instead, information communication with the outside through sensors in smart medical devices will enable the elimination of night patrols, the automation of care records, the facilitation of care through sleep data, and monitoring through behavioral data, which will influence the revision of required staffing standards.

Regarding the configuration of Managers, current standards require that the person in charge must be a full-time employee with knowledge and experience in older population’s care. However, under a smart care environment this may lead to resource inefficiency. The proposed revision adds the requirement that “the environment should ensure access to professional advice at any time,” while allowing smart technologies to reduce reliance on full-time personnel. The rationale is that technology can partially replace manual monitoring and management. The ultimate effect is to enhance institutional flexibility, relieve staffing pressures, and ensure that professional expertise is not compromised.

Regarding the configuration of Life advisors, existing standards stipulate a ratio of 100:1, with at least one full-time staff member. The revision maintains this ratio but emphasizes that at least one advisor must remain full-time, applying to both general and externally provided service facilities. The rationale is to ensure that the responsibilities of this core position are not weakened. The ultimate effect is to avoid excessive reliance on part-time staff, thereby maintaining the quality of care.

Regarding the configuration of Direct treatment staff, current standards determine ratios based on the number of residents (e.g., for 30 residents, a ratio of 50:1 with at least one full-time staff member). The revision introduces an additional clause allowing, when smart devices are deployed, for continuous operations to be maintained through nearby facilities or remote monitoring. The rationale is to leverage smart technologies to optimize staffing distribution. The ultimate effect is to mitigate shortages of direct care personnel while ensuring that care services and management functions are not compromised.

Therefore, it is foreseen that the use of smart devices will eliminate staffing shortages in fee-based nursing home, improve efficiency, reduce the physical burden on caregivers, and promote cooperation with medical caregivers as service housing that provides life support for the older population.

## 4 Discussion

The purpose of this study is to propose a revision framework for nursing home design standards based on smart care buildings. Through a review of existing regulations and standards (Phase I), an analysis of the demands of a super-aged society in conjunction with smart device applications (Phase II), and targeted improvement suggestions to current standards (Phase III), the study reveals that although existing regulations have established a relatively complete system in terms of accessibility, safety, and basic care efficiency, they remain insufficient in addressing the emerging needs brought by “smart care.”

In Phase I, the study systematically reviews the current architectural design, care service, and safety standards for nursing homes, comparing regulatory texts and industry guidelines to identify strengths and weaknesses and establish a baseline for subsequent research. In Phase II, building on these limitations, it incorporates the development trends of a super-aged society and advances in smart care technologies to analyze emerging demands at both architectural and service levels. The application of smart devices in health monitoring, environmental control, care assistance, and social support is highlighted as a way to address gaps in traditional standards, thereby linking social demand with technological possibility. In Phase III, the study proposes concrete revision strategies, including smart support for staffing configurations, the integration of digital accessibility into design, and the introduction of intelligent care equipment. This phase connects the shortcomings identified in Phase I with the future needs summarized in Phase II, ultimately providing actionable and integrable pathways for revising current standards.

The three phases form a progressive and interconnected logical chain: Phase I focuses on clarifying the framework and limitations of current standards, answering “where the system currently stands”; Phase II centers on the coupled demands of an aging society and smart care technologies, answering “what future development requires”; and Phase III builds on the first two phases to propose targeted revision pathways, answering “how institutional improvements can be realized.” In this way, the study constructs a systematic research pathway from diagnosing the existing framework to optimizing standards oriented toward smart care.

### 4.1 Innovation and practical significance

The innovation of this study lies first in its systematic comparison and identification of differences (Table 10). By conducting a structured comparison between Japan’s existing nursing home regulations and the standards required for “smart care buildings,” the study reveals significant gaps in areas such as digital support, personalized services, and flexibility in staffing. For example, in the domain of older population’s essential welfare services, current standards largely depend on in-person visits, periodic measurements, and caregiver operations, whereas the revised framework introduces smart devices and AI platforms to enable real-time health monitoring, personalized lifestyle counseling, smart household assistance, and intelligent nursing care, thereby effectively compensating for the limitations of traditional systems.

**Table 10:** Comparative differences between the revised and current standards.

Domain	Existing standards (current status)	Proposed revisions in this study	Main significance
Older Population essential welfare services	Rely on in-person visits, periodic measurements, and caregiver recordkeeping	Introduce smart devices to achieve real-time health tracking and emergency alerts	Improve safety, reduce reliance on manual labor, ensure timely response to emergencies
	Provide only basic health counseling, lacking personalized support	Use AI and remote platforms to deliver personalized health guidance and psychological support	Meet diverse needs of the older population, promote physical and mental well-being
	Household tasks mainly depend on caregivers or family members	Apply smart cooking, washing, and cleaning devices	Reduce physical workload, help the older population maintain autonomy
	Manual caregiver assistance for tasks such as bathing and toileting	Install smart toilets, adjustable bathing chairs, and temperature-controlled bathing systems	Enhance safety and efficiency of care, preserve dignity of the older population
Building design standards	Specify fixed installation heights for outlets and switches (e.g., bedside 80–90 cm, intercom 110 cm)	Remove certain rigid height requirements and adopt voice control, smart switches, etc.	Increase flexibility, adapt to diverse spatial layouts and older population users’ needs
Key points in end-of-life home care design	Family members must observe older population manually, limiting companionship	Introduce smart monitoring and communication devices	Enable families to monitor conditions in real time, extend companionship, enhance emotional connection
	Care efficiency depends on human labor; daily activities require large spaces; living room cannot serve both daily and reception functions	Use electric care beds, smart windows, and switches	Improve care efficiency, maintain daily and reception functions in compact spaces, enhance convenience and independence
	Privacy protection relies on physical partitions, difficult to adjust flexibly	Use smart windows and smart switches	Flexibly adjust privacy and spatial conditions, ensure safety and comfort
	Traditional models disrupt older adult’s lifestyle continuity	Apply smart monitoring and environmental sensing devices	Preserve original living habits, reduce discomfort caused by smart renovation
Staffing standards	Managers must have older population’s care knowledge and experience; nursing home directors must be full-time but may take on other duties if management is unaffected	Add requirement that “the environment should ensure access to professional advice at all times”; retain full-time requirement but allow smart environments to reduce reliance on full-time personnel	Enhance institutional flexibility, introduce smart support, alleviate staffing pressures
	Ratio of life advisors to users 100:1 or above, with at least one full-time staff member	Maintain the ratio, but emphasize at least one must be full-time; applies to both general and external service facilities	Ensure service quality, prevent excessive reliance on part-time staff that may weaken care
	Care staff ratios determined by number of residents (e.g., for 30 users, ratio of 50:1 with at least one full-time)	Add clause: “When smart devices are introduced, continuous operation can be maintained via on-site or nearby facilities, without interfering with nursing home management”	Use smart technologies to relieve labor shortages while ensuring care and management are not compromised

Second, the study proposes a revision framework. By mapping smart devices (such as sensors, data transmission systems, and operability-enhancing devices) against existing regulatory provisions, it develops a revision scheme covering three dimensions: welfare services, architectural design, and staffing standards. For architectural design, the study recommends abolishing rigid height requirements for certain outlets and switches and instead adopting voice control and smart switches to enhance spatial adaptability. For staffing standards, it suggests leveraging smart environments to alleviate labor shortages—for instance, by allowing smart monitoring to reduce the need for nighttime patrols—while ensuring service quality and improving staffing flexibility.

Finally, the study emphasizes operability and policy transferability. The proposed revisions are not confined to theoretical discussions but are designed for practical application. For example, measures such as “abolishing mandatory height requirements for outlets,” “reducing the need for nighttime patrols,” and “introducing smart toilets and temperature-controlled bathing systems” can be readily embedded in regulatory provisions and architectural practice. The shared goal of these revisions is to improve safety and efficiency, meet the diverse and personalized needs of the older population, relieve labor shortages in caregiving, and at the same time preserve the dignity and autonomy of older population.

### 4.2 Limitations and future work

It should be acknowledged that the revision proposals in this study are mainly based on commonly available and relatively mature smart devices on the market, such as wearable monitors, smart windows, and voice-interaction systems. This device-oriented approach inevitably entails limitations, as its applicability and forward-looking capacity are constrained by the current level of technological development. With the continuous iteration of smart care technologies, the emergence of new devices and systems may place additional requirements on existing design and staffing standards.

Moreover, the proposed revisions are primarily derived from theoretical analysis and comparative review of standards, lacking systematic validation in actual nursing home environments. This implies that, although the findings are logically sound and operationally feasible, their applicability across different regions, facility types, and older populations still requires further testing.

Future research may proceed in two directions. First, it should continuously monitor the development of smart care devices and systems, promoting dynamic revisions of regulatory standards to keep pace with technological progress. Second, it should conduct field-based case studies and experimental applications to verify the feasibility and effectiveness of the proposed revisions, thereby providing more solid evidence to support policymaking and industry adoption.

## 5 Conclusion

In this study, proposals are made to optimize existing regulations and standards in Japan based on the use of smart devices in nursing home.

This study first reviewed housing standards and regulations for older population’s housing in Japan and by collating related standards and regulations, research team aimed to analyze and summarize the requirements for older population’s housing met by existing standards and regulations on the one hand, and on the other hand, to use the results of the collation as a basis for subsequent optimization. Also in this section, the importance of optimizing existing regulations is made clear, and the need to enable the introduction of smart devices to interface with existing regulations while improving their relevance and adaptability to better serve the older population.

Next, smart devices were surveyed and evaluated. Based on the summarized regulations and housing requirements for the older population, research team referenced the Japanese public awareness survey and the smart medical devices to be introduced. The aim was to clarify the characteristics of these devices and provide a basis for optimizing existing regulations to align with these smart devices. In this section, the study proposes that the integration of smart devices not only helps to improve the efficiency of care, but also reduces the physical burden on caregivers, while facilitating collaboration among healthcare professionals through smart means.

Finally, the selected smart medical devices are combined with nursing home, and the influence on regulations and standards, and the improvement of the content of regulations due to their effects are clarified according to the functions of the smart devices used. This study focuses on the common smart devices currently on the market, and subsequently, as new devices are proposed, the relevant standards can also be adjusted along the lines of this paper.

The regulation content revision, from the point of view of care places and caregivers, can eliminate the problem of shortage of staff for the older population with fee-based nursing home, improve efficiency, reduce the physical burden of caregivers, and promote cooperation with medical caregivers; for the older population, it can reduce the barriers to the operation of facilities and services for the older population while satisfying the demands of the older population of the next generation.

## Data Availability

The original contributions presented in the study are included in the article/supplementary material, further inquiries can be directed to the corresponding author.
